# Predicting liver SBRT eligibility and plan quality for VMAT and 4π plans

**DOI:** 10.1186/s13014-017-0806-z

**Published:** 2017-04-24

**Authors:** Angelia Tran, Kaley Woods, Dan Nguyen, Victoria Y. Yu, Tianye Niu, Minsong Cao, Percy Lee, Ke Sheng

**Affiliations:** 1 Department of Radiation Oncology, University of California, 200 Medical Plaza, Suite B265, Los Angeles, CA 90095 USA; 2 Translational Medicine Institute, Zhejiang University, Zhejiang, China

## Abstract

**Background:**

It is useful to predict planned dosimetry and determine the eligibility of a liver cancer patient for SBRT treatment using knowledge based planning (KBP). We compare the predictive accuracy using the overlap volume histogram (OVH) and statistical voxel dose learning (SVDL) KBP prediction models for coplanar VMAT to non-coplanar 4π radiotherapy plans.

**Methods:**

In this study, 21 liver SBRT cases were selected, which were initially treated using coplanar VMAT plans. They were then re-planned using 4π IMRT plans with 20 inversely optimized non-coplanar beams. OVH was calculated by expanding the planning target volume (PTV) and then plotting the percent overlap volume *v* with the liver vs. *r*_*v*_, the expansion distance. SVDL calculated the distance to the PTV for all liver voxels and bins the voxels of the same distance. Their dose information is approximated by either taking the median or using a skew-normal or non-parametric fit, which was then applied to voxels of unknown dose for each patient in a leave-one-out test. The liver volume receiving less than 15 Gy (V_<15Gy_), DVHs, and 3D dose distributions were predicted and compared between the prediction models and planning methods.

**Results:**

On average, V_<15Gy_ was predicted within 5%. SVDL was more accurate than OVH and able to predict DVH and 3D dose distributions. Median SVDL yielded predictive errors similar or lower than the fitting methods and is more computationally efficient. Prediction of the 4π dose was more accurate compared to VMAT for all prediction methods, with significant (*p* < 0.05) results except for OVH predicting liver V_<15Gy_ (*p =* 0.063).

**Conclusions:**

In addition to evaluating plan quality, KBP is useful to automatically determine the patient eligibility for liver SBRT and quantify the dosimetric gains from non-coplanar 4π plans. The two here analyzed dose prediction methods performed more accurately for the 4π plans than VMAT.

## Introduction

Stereotactic Body Radiation Therapy (SBRT) has emerged as a promising treatment modality for hepatocellular carcinoma (HCC), liver oligometastatic disease and other liver tumors when patients are contraindicated for surgical resection [1, 2]. Compared to conventionally fractionated radiotherapy, the hypofractionated SBRT treatment, delivered in 3–5 fractions, is more biologically effective to achieve improved local control rates [2–4]. Brown et al. [5] attributed the success of SBRT to dose escalation enabled by improved dose conformality and delivery accuracy using modern planning and delivery techniques, including intensity modulated radiotherapy (IMRT) and image guided radiation therapy. It has been recently shown that by automatically selecting and optimizing non-coplanar beams in the 4π planning method, the dose conformality can be further improved to spare a larger volume of normal tissue from high dose irradiation [6, 7]. However, the dosimetric improvement may come at a cost of substantially prolonged treatment time due to the time to set up the number of non-coplanar couch angles. On the other hand, even with these technological advances, the patient eligibility for a specific treatment method due to critical organ tolerance to the aggressive dose fractionations is unclear. The liver volume doses, among other metrics, have been shown to correlate with radiation-induced liver disease [8, 9].

In an earlier study to provide guidelines to treatment planning, a predictive model was developed to calculate the maximum tolerable dose for Helical TomoTherapy-based SBRT [10]. The model provided a crude estimate for the achievable target dose based on the liver and PTV volume but it did not consider the relative position of the PTV to the liver and was unable to predict the liver dose volume for a given prescription. In practice, a trial and error planning process is adopted to determine the level of normal organ doses and patient eligibility but the result can be operator and planning method dependent. As a result, potentially eligible patients may be turned down for SBRT or treated to a reduced and less effective dose [11]. A recently developed paradigm referred to as knowledge based planning (KBP) may be helpful to improve the process.

KBP was developed to address the challenge that even with the guidance of published protocols, the planning goals for an individual patient are unclear. This ambiguity may lead to either wasted planning time on plans that cannot be further improved or stopping prematurely when a better plan quality can be attained [12–15]. KBP attempts to overcome this challenge by developing geometric and dosimetric associations with previously treated patients and using these associations to predict the dosimetry of a new patient. There have been numerous studies using KBP to identify suboptimal plans with unnecessary dose to critical structures [12–17]. Specifically for fractionated liver radiotherapy, a commercial KBP tool has been used to automatically create planning objectives that resulted in clinically acceptable plans [18].

In the current study, we aim to answer a different set of questions for the liver SBRT planning regarding the accuracy of two popular KBP methods to predict coplanar VMAT and non-coplanar 4π radiotherapy plan dosimetry. Answering these questions will elucidate not only the predictability of the two different planning methods, without going through the lengthy planning process, but also the potential gains of using the time consuming 4π radiotherapy for specific patients.

## Methods and materials

### Patients and volumetric modulated arc therapy (VMAT) planning

Twenty-one consecutive liver SBRT patients were selected with prescription doses ranging from 30 to 60 Gy in five fractions. Liver volumes ranged from 550 to 3346 cc and PTV volumes from 2 to 222 cc. The patient cohort includes a pediatric patient with a small liver volume and a patient with enlarged liver due to cancer infiltration. The patients were clinically treated using VMAT (RapidArc, Eclipse Treatment Planning System version 10, Varian) with either 2 coplanar full arcs or 2–3 coplanar partial arcs entering the body proximal to the tumor, depending on the tumor laterality. The VMAT optimization and dose calculation in Eclipse used the Progressive Resolution Optimizer (PRO, version 10.0.28) and anisotropic analytical algorithm (AAA, version 10.0.28) with 2.5x2.5x2.5 mm^3^ grid resolution, respectively. Collimator rotations were used between arcs. All plans were normalized to cover 95% of the PTV by the prescription dose. For the OARs, plan objectives are liver volume receiving less than 15 Gy > 700 cc, stomach and bowel volumes receiving more than 20 Gy < 20 cc, maximal dose to kidney and spinal cord <12Gy. The same objectives were applied to all prescription doses.

### 4π radiotherapy

The 21 liver patients were re-planned using 4π radiotherapy [6, 7]. 4π radiotherapy utilizes a candidate pool of 1162 non-coplanar beams evenly distributed 6° apart throughout the 4π steradian solid angle space. The treatment geometry was predicted based on a computer assisted design (CAD) model of a Varian TrueBeam machine and a 3D scan (Artec 3D camera) of a human subject [19]. Beams resulting in collision between the gantry and couch or patient were eliminated. An in-house greedy column generation algorithm was used to iteratively select and optimize 20 non-coplanar beams from the candidate pool [20, 21]. The column generation process chooses each optimal beam one at a time and performs fluence map optimization after every beam, which is necessary to choose the subsequent optimal beam. The gantry and couch angles for the 20 4π beams were then imported into Eclipse to reoptimize the fluence maps and calculate the final IMRT dose using Dose Volume Optimizer (DVO, version 10.0.28) and AAA algorithms. The dose calculation algorithms and parameters are identical to those used in VMAT plans, allowing unbiased comparison between the *clinically feasible* 4π and the VMAT plans. When the 4π beam angles were imported into the Eclipse planning system, they were converted from the patient centered steradian angles to gantry and couch angles.

### Predicting liver dose distribution using KBP

Based on a multi-institutional phase I/II liver SBRT trial, the liver volume receiving 15 Gy or less was used as the eligibility criterion for SBRT treatment [4]. A minimum of 700 cc liver tissue should be spared from 15 Gy. The criterion was derived based on surgical and conventional fractionated radiation therapy experience and confirmed by subsequent clinical trials [4]. The patient with small liver volume was an exception that did not meet the criterion. However, this did not influence our study predicting the liver V_<15Gy_. Two methods, overlap volume histogram (OVH) [16] and statistical voxel dose learning [13] were adapted to establish the correlation between patient geometry and dose. Both KBP methods used the OAR and dose data at 2.5x2.5x2.5 mm^3^ resolution. The various KBP methods and their prediction capabilities are summarized in Table 1 and described in the following text. All KBP methods were used to predict both VMAT and 4π dose.

**Table 1:** Summary of KBP methods and prediction capabilities

Treatment planning method	OVH	SVDL		
		Median	Non-parametric	Skew-normal
4π	V_<15Gy_	V_<15Gy_, DVH, 3D dose	V_<15Gy_, DVH	V_<15Gy_, DVH
VMAT	V_<15Gy_	V_<15Gy_, DVH, 3D dose	V_<15Gy_, DVH	V_<15Gy_, DVH

#### Predicting liver dose volume values using OVH

The OVH is defined, as described by Wu et al. [16, 22], as the fractional volume of the OAR volume (V_O_) within distance *r* of the target (*T*)

$$ \mathrm{O}\mathrm{V}\mathrm{H}(r)=\frac{\left\{ p\in {V}_O\Big| d\left( p, T\right)\le r\right\}}{V_O} $$

where *d(p,T)* is the signed distance between point *p* and the target boundary such that *d* is negative within the target and positive outside. This is physically equivalent to isotropically expanding the target by *r* cm and calculating the fractional volume of the OAR that overlaps with the expanded target. When the OAR overlaps with the PTV, the target needs to be contracted until there is no overlap to obtain the complete OVH.

A single point from the OVH: *r*_*v*_ *,* the expansion distance to overlap fractional volume *v,* was used to quantify the relationship between the OVH and the liver volume receiving less than 15 Gy, denoted by V_<15Gy_ [16]. The correlation coefficients of various *r*_*v*_ values with liver V_<15Gy_ were evaluated, including *v* = 5, 10, 15, 20, and 30%. Linear regression was used to calculate and predict the liver V_<15Gy_ from the *r*_*10*_ of an unplanned patient.

#### Predicting liver dose using statistical voxel dose learning (SVDL)

The minimal Euclidean distance from each OAR voxel to the target is defined as

$$ m\left( p, T\right) = \underset{t\ \in\ T}{ \min}\left| p- t\right| $$

where *p* ∈ V_O_ and T is the subset of target boundary voxels. The voxels were sorted into bins based on their distance to the target. There will then be a resulting histogram of doses received by voxels of the same distance bin. SVDL is the method of utilizing this distance-dose information to predict dose.

To approximate the dose distribution of each distance bin and then apply the result to liver voxels of a patient with unknown dose, three sub-approaches were tested. First, a single median value was used to represent the distribution. The distribution mean and mode were also tested using this method, but were not found to be as accurate as using the median to approximate the distribution. The approach clearly loses information but can assign a single dose value to each voxel of a new patient, allowing it to predict liver V_<15Gy_, DVH, and 3D dose. Second, to capture more statistical information from the distribution, two algorithms were applied to correct the bias in the raw data sampling due to fewer available points for voxels at a shorter distance to the PTV. A skew-normal distribution was used to fit the raw distribution as described by Appenzoller et al. [13]. Alternatively, the distribution was resampled by convolution with a Gaussian kernel so that each distance bin was represented by an equal number of 10,000 data points.

#### Evaluating prediction accuracy

The performance of OVH and SVDL in predicting liver V_<15Gy_, DVH, and 3D dose was evaluated and compared using a leave one out cross validation (LOOCV) test. For each patient, it is “left out” of the training set, which is then used to predict the patient’s dose using either OVH or SVDL. V_<15Gy_ prediction error was evaluated as the percent error defined as:

$$ Percent\  error = \frac{\left| Actual- Predicted\right|}{Actual}\times 100. $$

Additionally, for the SVDL method, prediction of the entire liver DVH was evaluated using the residual sum of squares (RSS), which is defined as

$$ R S{S}_{D V H} = {\displaystyle \sum_{D=0}^{\infty }}{\left(\left( D V{H}_{actual}(D) - DV{H}_{predicted}(D)\right) \cdot \varDelta D\right)}^2. $$

Low RSS values indicate good agreement between the actual and predicted DVH throughout the whole DVH.

Likewise, the residual can be used to evaluate the predictive accuracy of the 3D dose distribution, normalized to the number of voxels making the mean squared error (MSE), for the single value SVDL prediction method,

$$ M S{E}_D = \frac{{\displaystyle {\sum}_{i = 1}^n}{\left({D}_{i, actual} - {D}_{i, predicted}\right)}^2}{n}. $$

where n is the number of voxels in the OAR.

Because of the narrower 4π dose-distance spread, a paired one-tailed t-test was performed to compare the prediction error of VMAT and 4π plans. Results were considered significant if *p <* 0.05. Calculations were performed using MATLAB (version R2015a, MathWorks) on a PC with two Intel Xeon E5 processors at 3.10 GHz.

## Results

### OVH

**Fig. 1:**
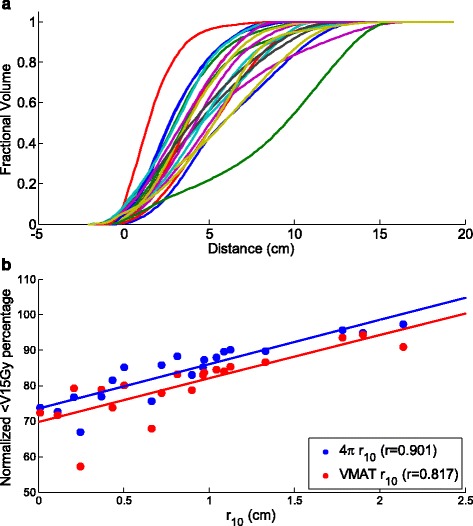
**a** OVH for all 20 patients. **b** Expansion distance at 10% OVH vs. percentage of liver receiving less than 15 Gy

Figure 1a shows the liver OVH of all 21 patients for 4π and VMAT plans and Fig. 1b shows the correlation between *r*_*10*_ and the liver volume receiving less than 15 Gy. *r*_*10*_ showed the best correlation, with correlation coefficients of 0.897 and 0.815 for 4π and VMAT liver V_<15Gy_, respectively, and was used for subsequent analysis. Using a least-squares linear fit, the *normalized* liver V_<15Gy_ is correlated with *r*_*10*_ by:

$$ {V}_{<15 Gy}^{4\uppi}=14.2{r}_{10}+71.8 $$

$$ {V}_{<15 Gy}^{V MAT}=15.0{r}_{10}+67.1 $$

for the 4π and VMAT plans, respectively. The results suggest that on average, 4π therapy increased liver V_<15Gy_ by 4.7%, corresponding to an average volume increase of 78.9 cc, which shows an improvement in the 4π planning technique compared to the previous publication [6]. The percent error in the LOOCV test predicting the liver V_<15Gy_ using OVH *r*_*10*_ is shown on the two leftmost columns of Fig. 3a) as a boxplot of the results from the 21 patients.

### SVDL

**Fig. 2:**
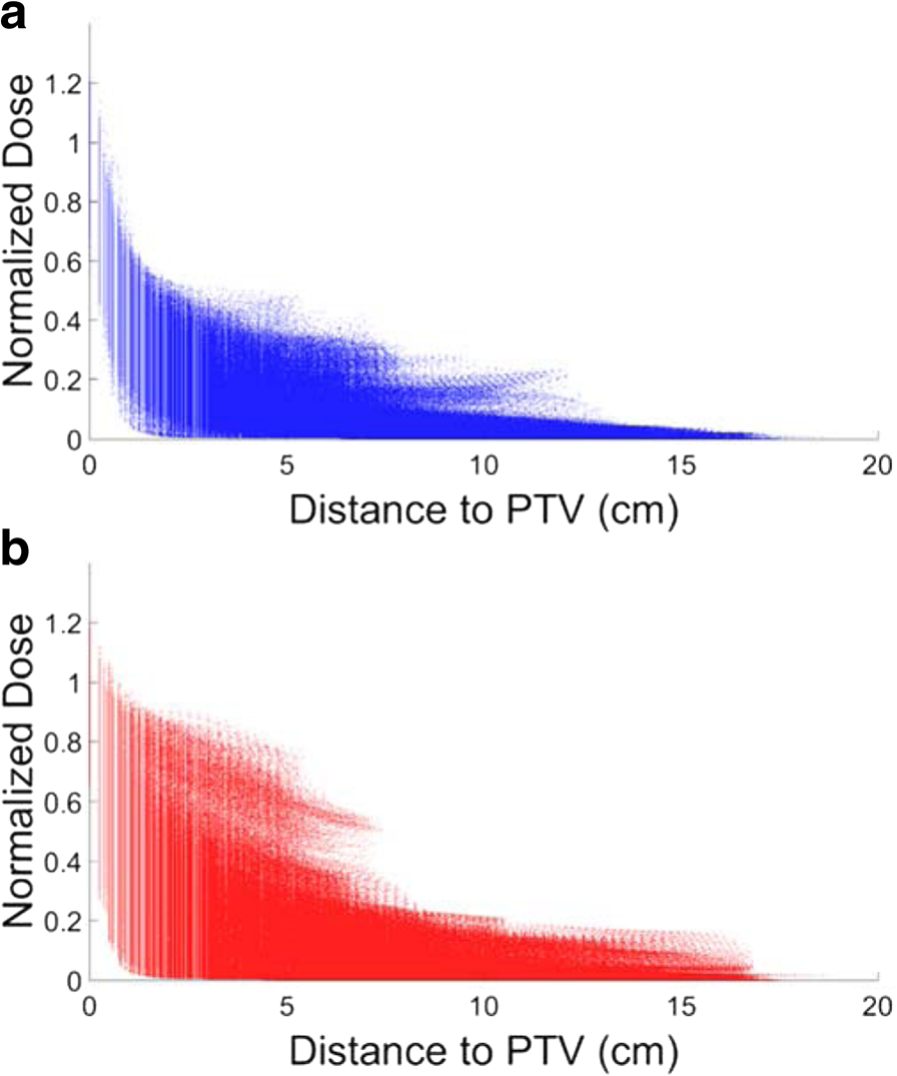
Distance to the target vs. dose received at every liver voxel for **a** 4π and **b** VMAT plans

**Fig. 3:**
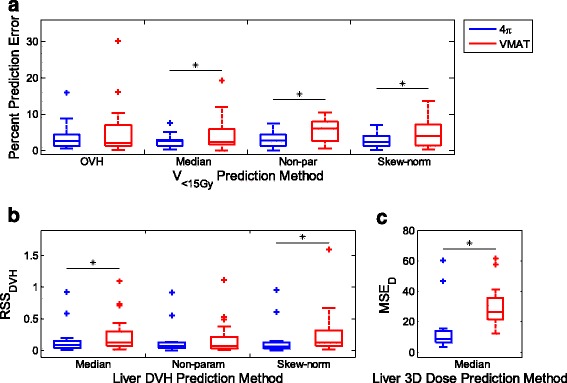
Boxplot results for the leave-one-out cross validation tests for the 21 patient cohort, where median, non-parametric, and skew-normal are the SVDL methods. For each box, the central mark represents the median and the edges the 25th and 75th percentiles. Whiskers cover the remaining data not deemed outliers, *p < 0.05. **a** Percent error in predicting the liver V < 15Gy using OVH and SVDL for 4π and VMAT plans. **b** Residual sum of squares analysis using SVDL to predict 4π and VMAT DVHs. **c** Residual sum of squares analysis for predicting the 3D dose wash using median SVDL prediction for 4π and VMAT

Figure 2a, b shows a scatter plot of the distance to the target vs. dose received for every liver voxel from the patient cohort for 4π plans and VMAT plans. Each column of data points represents the data within each distance bin. The percent error in predicting the liver V_<15Gy_ of the patient cohort is presented in Fig. 3a, with 4π and VMAT represented in blue and red, respectively. Compared to SVDL, OVH resulted in the worst V_<15Gy_ prediction performance for 4π with an average percent error of 3.76%, compared to 2.58˗2.97% for the SVDL prediction methods. OVH also resulted in an average percent error of 5.33% for VMAT, which is superior to SVDL using non-parametric fitting but inferior to other SVDL methods.

**Fig. 4:**
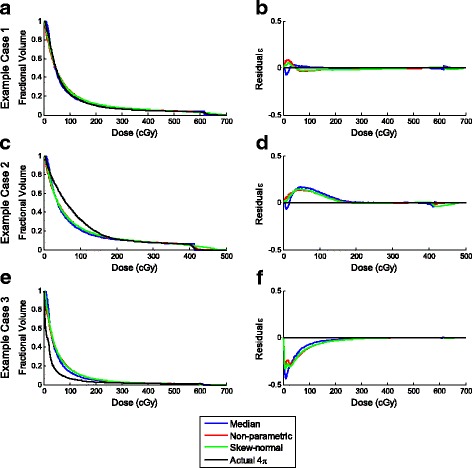
**a**, **c**, **e** DVHs and **b**, **d**, **f** residuals of the various SVDL prediction methods for three example 4π cases

**Fig. 5:**
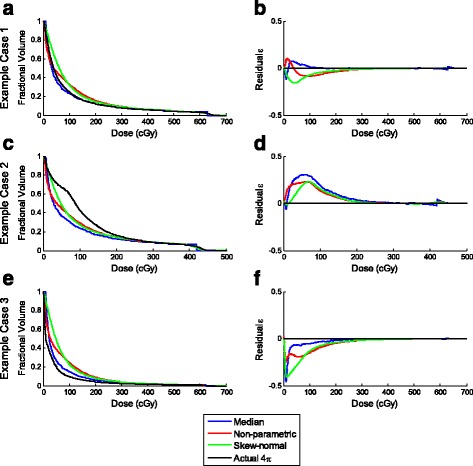
**a**, **c**, **e** DVHs and **b**, **d**, **f** residuals of the various SVDL prediction methods for three example VMAT cases

Boxplots of the RSS_DVH_ for the patients are shown in Fig. 3b. The actual and predicted DVHs along with their corresponding residuals for three distinctive cases are shown in Figs. 4 and 5, for 4π and VMAT plans respectively. Here, the predicted DVHs for all SVDL methods – median approximation, non-parametric, and skew-normal fitting prediction – are shown. The first example (top rows) shows a DVH that was accurately predicted by most prediction methods, so its corresponding residual is close to zero. The second (middle rows) and third (bottom rows) examples are DVHs that were under- or over-predicted.

The SVDL method using the median of each distance bin to predict the liver V_<15Gy_ resulted in the lowest average prediction error for both 4π and VMAT plans. The non-parametric and skew-normal fitting methods did not improve the liver V_<15Gy_ prediction despite more statistical information being retained. In contrast, although the median SVDL proved to be better at predicting V_<15Gy_, the SVDL methods retaining more statistical information were better at predicting DVHs. In this case, as shown in Fig. 3b, the non-parametric fitting SVDL method attained the lowest average RSS_DVH_ for both 4π and VMAT plans, followed by the skew-normal and median SVDL methods. In other words, keeping the statistical information has a slight benefit in predicting the DVH.

In addition to the differences in performance resulting from using two different KBP methods, another important finding is the predictability of the VMAT and the non-coplanar 4π plans. The V_<15Gy_ prediction accuracy of 4π plans was consistently superior to the VMAT plans using all prediction methods. The differences in V_<15Gy_ prediction error were statistically significant between 4π and VMAT for all prediction methods. The same conclusion applies to RSS_DVH_; 4π prediction was shown to be more accurate than that of VMAT, producing statistically lower errors for skew-normal SVDL.

**Fig. 6:**
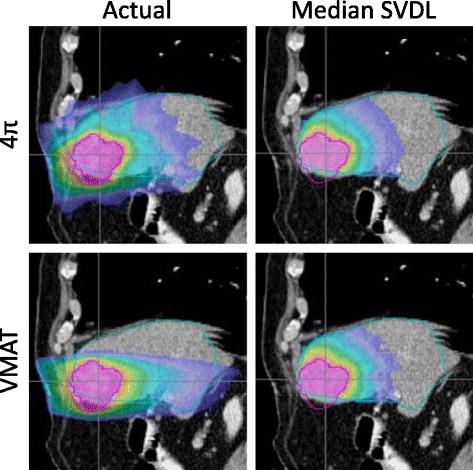
Actual and predicted dose for a sagittal slice of an example patient using the median SVDL prediction method for 4π and VMAT. While the actual dose includes dose for the whole body, SVDL only predicts dose within the liver

The difference in predictability of VMAT and 4π plans can be intuitively appreciated in Fig. 2 and Fig. 6. Figure 2 shows greater dose spread for voxels at the same distance to the PTV in the VMAT plans and clear clusters indicating certain geometrical heterogeneity. Figure 6 shows the achieved and predicted liver dose for a sagittal slice of an example patient, using the median SVDL prediction method for both 4π and VMAT. SVDL predicted a more isotropic dose distribution that better matched the 4π dose distribution than that of VMAT, which showed significantly steeper dose gradient in the superior/inferior direction than the anterior/posterior and medial/lateral directions. The difference in prediction accuracy is quantitatively reflected in the MSE_D_ (Fig. 3c). Median SVDL resulted in relatively low MSE_D_ for 4π and VMAT 3D dose prediction. Quantitatively, the 3D dose of the non-coplanar 4π plans were more accurately predicted than VMAT, attaining significant results for all applicable prediction methods. The average 4π MSE_D_ was less than half of the average VMAT MSE_D_ using the same prediction method.

Total training and prediction times are listed in Table 2. Calculating OVH for each patient usually takes 1–2 min, but *r*_*v*_ calculation and correlation are fast once OVHs are calculated for all patients. OVH is considerably faster than SVDL, which acquires geometric information by calculating the minimal Euclidean distance to the PTV for every liver voxel of every patient. This typically takes 1–2 min depending on the size of the liver. However, unlike OVH prediction, SVDL requires calculation of the distance bins taking and additional 1–2 min, which needs to be redone for every training set. On top of the distance bin organization, non-parametric and skew-normal SVDL take significantly longer time to fit each distance bin than simply taking the median. Once training is finished, liver dose predictions can be made for any subsequent test patient as fast as 1 s for OVH and median SVDL or around 5 min for non-parametric and skew-normal fitting SVDL.

**Table 2:** Total training and prediction time per training set

		Calculation time per training set	
		Training time	Prediction time
OVH		1–2 min	~1 sec
SVDL	Median	2–4 min	~1 sec
	Non-parametric	~1 hour	~5 min
	Skew-normal	~1 hour	~5 min

## Discussion

Several highly relevant questions in liver SBRT are investigated in the study. The first is whether it is feasible to predict the patient eligibility for SBRT considering the sensitivity of normal liver tissue to radiation dose. The second is which of the two available methods is better to model and predict the liver dose. The third question is whether the gain in dose compactness by using non-coplanar radiotherapy is meaningful for a specific patient.

To answer these questions, our study employed two state of the art KBP methods to predict the liver volume receiving less than 15 Gy as well as the full DVH and 3D dose for coplanar VMAT and non-coplanar 4π radiotherapy. The study shows that, using the fully automated methods, the liver V_<15Gy_ can generally be predicted with errors lower than 5%. Between the two KBP methods, OVH is less accurate than SVDL but faster because the OVH curve only needs to be calculated once. However, the time benefit comes at a cost of limited information available from OVH. For example, only specific metrics like V_<15Gy_ can be extracted from OVH data. By adding 1–2 min per patient for the SVDL method, the full DVH and 3D dose can be predicted. Within SVDL, the remarkable performance displayed by fast median value method relative to the other metrics makes it the preferred method for quick and accurate dose prediction.

More interestingly, in addition to quantifying a systematic advantage, the study reveals a previously unreported advantage of using optimal non-coplanar geometry for liver SBRT, which is the superior predictability of the non-coplanar liver plans using KBP based on Euclidian distances *alone*. Both coplanar VMAT and the non-coplanar 4π plans are independent on the operator to manually specify the beam orientations, the difference in prediction accuracy can be intuitively understood by comparing the isotropicity (Fig. 6).

Previous KBP studies involving coplanar radiotherapy have either disregarded the anisotropic aspects of the dose or empirically divided the voxels into in-plane and out-of-plane voxels based on their relative superior/inferior location to the PTV [23]. However, it is important to note that there are several pitfalls modifying the dose prediction methods to include non-isotropicity. First, because of beam divergence, even without intensity modulation, VMAT dose distribution cannot be simply described by a cylinder with defined inferior and superior boundaries. Second, the geometric dose distribution is further complicated by intensity modulation for individual patients. Third, 4π dose distribution is more isotropic than VMAT but still far from a perfect sphere due to the elongated human body shape and finite number of non-coplanar beams. In practice, the empirical inclusion of beam geometry in the Euclidian distance did not improve the statistical spread [12]. Since there is no simple universal way to include this non-isotropicity in prediction without relying on heuristics that may weaken the robustness of prediction, we believe the comparison using simple methods in the study provide is still fair and informative.

Despite the reduced prediction error for the 4π plans, there is still a residual statistical spread of voxel doses at the same distance to PTV, as seen in Fig. 2. It is important to understand the causes of the remaining uncertainties, as they are partially responsible for residual errors observed in the downstream liver V_<15Gy_, DVH and isodose predictions. The actual dose distribution largely depends on how the beams are arranged, which being planning variables are not included as prediction parameters. The discretized beams resulted in non-smooth low dose fall-off that is distinctly different from the smooth dose fall-off predicted based on Euclidean distances alone. However, not all discrepancies can or should be eliminated. Factors including patient, PTV, and liver sizes, as well as the presence of nearby OARs should be modeled and separated from the inconsistent plan quality, which should then be ultimately minimized. While these factors affect the accuracy of the methods reported in this study, if they can be incorporated into the KBP prediction technique, they should help decrease the prediction errors.

An appealing opportunity here is the potential of fully automating treatment planning. In a commercial treatment planning system, KBP for VMAT has enabled automated generation of DVH objective points and then clinically acceptable plans [18]. Similarly, 4π plans have the potential to be fully automated if the plan quality can be predicted for a new patient to guide the automated planning process. We will study the additional complexity from beam orientation optimization not in the VMAT planning process.

## Conclusion

Two existing KBP methods are used to predict the liver dose of liver SBRT patients using coplanar VMAT or non-coplanar 4π planning methods. Between the two compared KBP methods, SVDL was more accurate than OVH in all cases. Within SVDL, voxel dose estimated using the computationally inexpensive median value performed similar to or better than the SVDL methods retaining the complete statistical information. The best method depends on the goal of the prediction. For liver SBRT eligibility, our results show that the liver volume receiving 15 Gy or less can be predicted within 5%, which is most accurate with median value based SVDL. We also showed that compared to the VMAT method, 4π plan dosimetry can be more accurately predicted. Because the improved liver sparing using non-coplanar 4π radiotherapy was also reflected in the model, KBP provides an individualized prediction of the benefit using the more complex 4π radiotherapy for liver SBRT. The information can be used to aid choice of treatment strategies.

## Abbreviations

AAA: Anisotropic analytical algorithm
CAD: Computer assisted design
DVH: Dose volume histogram
HCC: Hepatocellular carcinoma
IMRT: Intensity-modulated radiation therapy
KBP: knowledge based planning
LOOCV: leave one out cross validation
MSE: mean squared error
OAR: Organ-at-risk
OVH: Overlap volume histogram
PTV: Planning target volume
RSS: residual sum of squares
SBRT: Stereotactic body radiotherapy
SVDL: Statistical voxel dose learning
VMAT: Volumetric-modulated arc therapy
